# A single-nucleotide polymorphism (rs1805087) in the methionine synthase (METH) gene increases the risk of prostate cancer

**DOI:** 10.18632/aging.101584

**Published:** 2018-10-18

**Authors:** Xiaosong Zhang, Jilei Tang, Nan Shen, Kewei Ren

**Affiliations:** 1 Department of Urology, Nantong Tongzhou District People’s Hospital, Nantong 226300, China; 2 Department of Orthopedics, Qidong People’s Hospital, Nantong 226200, China; 3 Department of Clinical Pharmacy, the Affiliated Jiangyin Hospital of Southeast University Medical School, Jiangyin 214400, China; 4 Department of Orthopedics, the Affiliated Jiangyin Hospital of Southeast University Medical School, Jiangyin 214400, China

**Keywords:** methionine synthase, polymorphism, prostate cancer, susceptibility, meta-analysis

## Abstract

Methionine synthase (METH, i.e., MTR) is a key enzyme in the folate pathway, which plays a critical role in the synthesis, repair, and methylation of DNA. The association between *METH* gene polymorphisms and prostate cancer susceptibility remains ambiguous. Thus, we performed an updated meta-analysis of *METH* single-nucleotide polymorphism rs1805087 involving 12 independent case-control studies comprising 9986 prostate cancer patients and 40134 controls. The odds ratio and 95% confidence intervals were applied to evaluate the relation of this single-nucleotide polymorphism with prostate cancer. Statistical analysis was performed in STATA 11.0. A significant association was found between rs1805087 and increased prostate cancer risk, overall and with Hardy–Weinberg equilibrium. In subgroup analyses (based on ethnicity, source of control, genotyping methods, or publication status), similar associations were observed (e.g., genotype GA vs. AA: odds ratio 1.19, 95% confidence interval 1.01–1.40 among whites; G allele vs. A allele: odds ratio 1.14, 95% confidence interval 1.02–1.28 among hospital-based controls). Thus, the common polymorphism (rs1805087) of *METH* may be associated with increased prostate cancer risk. Further studies with a larger sample size and detailed gene–environment interactions should be conducted to identify the role of *METH* polymorphisms in prostate cancer susceptibility.

## Introduction

Prostate cancer (PCa) is a leading cause of cancer-associated deaths among men in developed countries [1]. Although the incidence of PCa is much lower in the Asian population than in its western counterpart, in recent years, the incidence of PCa and the PCa mortality rate in some Asian countries grew rapidly [2,3]. Age, a positive family history, African-American origin, alcohol use, and certain gene mutations are significant risk factors of PCa [4,5]. Particularly, genetic factors play a prominent role in the pathogenesis of PCa. For example, Lynch syndrome (germline mutations in *MLH1*, *MSH2*, *MSH6*, *PMS2*, or *EPCAM*) increases the risk of PCa two- to fivefold, in comparison with the general population [4,6–8].

Folate metabolism–associated genes involved in both DNA methylation and repair are thought to play an important part in tumorigenesis [9]. The gene encoding methionine synthase (METH, i.e., MTR), a key enzyme in the folate pathway, is located on human chromosome 1 (1q43) [10,11]. Encoded by 34 exons, the METH protein is 1265 amino acid residues long, with a molecular weight of 140.5 kDa [12]. A common polymorphism in *METH* has been found at position 2756 (METH A2756G; rs1805087) [13]. This polymorphism may promote homocysteine upregulation and DNA methylation, thereby increasing the enzymatic activity of METH [14]. Moreover, this polymorphism may result in CpG island hypermethylation in tumor suppressor genes, such as *TP53* [15].

Some studies that have addressed the effects of the *METH* rs1805087 single-nucleotide polymorphism (SNP) on the risk of PCa have yielded inconsistent results. Collin et al. (2009) [16] investigated the effect of eight SNPs, including *MTHFR* rs1801133, *MTHFR* rs1801131, *METH* rs1805087, *METHR* rs1801394, *MTHFD1* rs2236225, *SLC19A1/RFC1* rs1051266, *SHMT1* rs1979277, and *FOLH1* rs202676, on PCa risk in a meta-analysis and found no significant effects of any of these SNPs on PCa susceptibility. The study included eight case-control studies (five of which have not been published) on rs1805087. Since 2009, many more original research articles have been published, making it necessary to combine the data from all the aforementioned studies and reanalyze the association between rs1805087 and PCa susceptibility.

The objective of our study was to examine associations between rs1805087 genotypes and the risk of PCa in larger samples by meta-analysis (9986 PCa patients and 40134 controls from 12 studies) [16–27].

## RESULTS

### Study characteristics

**Figure 1:**
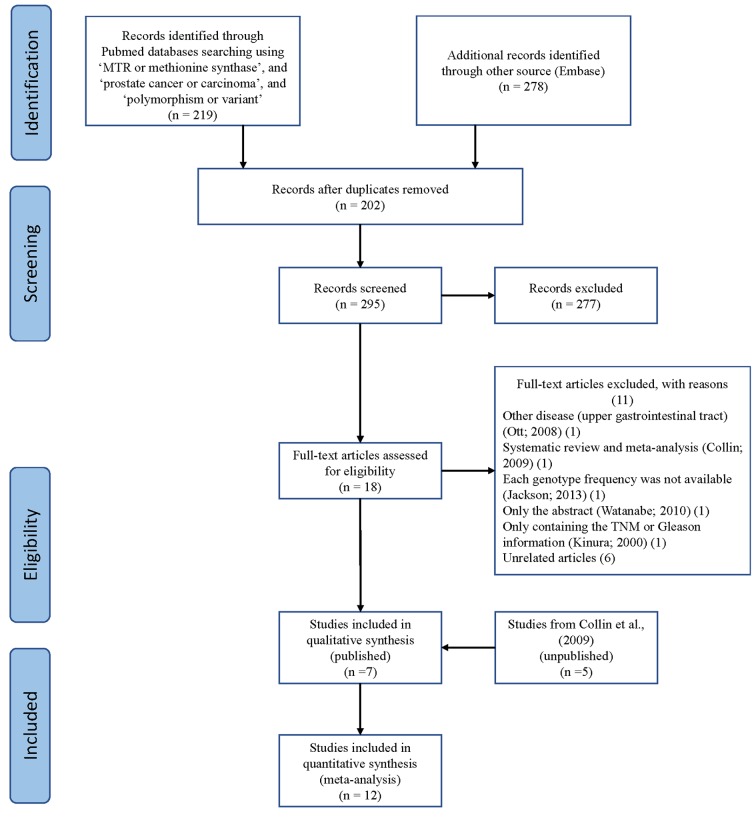
**A flowchart illustrating the search strategy used to identify studies on association of *METH* SNP rs1805087 with PCa risk**.

Using different combinations of key terms, 24 studies were identified in PubMed and Embase. As shown in Figure 1, seven duplicate studies were excluded. Among the remaining 17 studies, five were excluded: one about the upper gastrointestinal tract [28], one meta-analysis [16], one abstract [29], one involving TNM or Gleason scores [30], but lacking genotype frequencies of cases/controls, and one missing the available genotype frequencies [31]. We also included the study by Collin et al. (2009), which contains findings from five unpublished studies [16,17,19,22,24,26].

**Figure 2:**
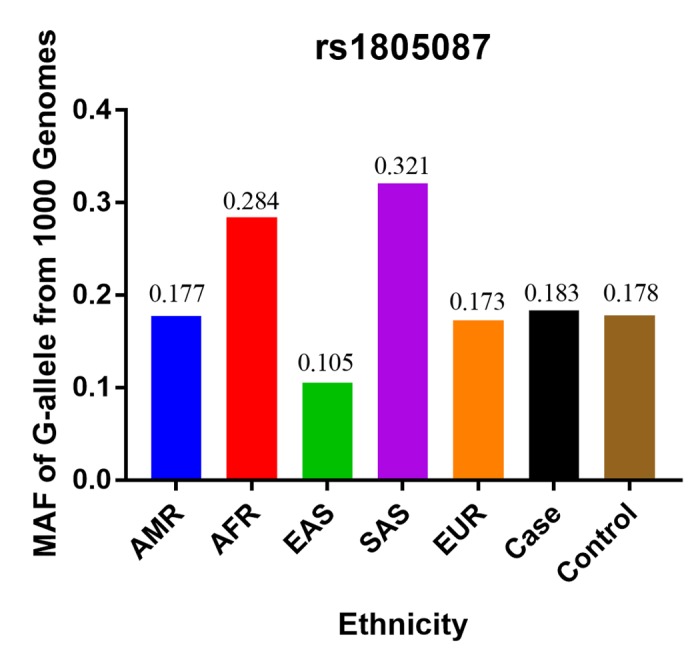
**The MAF of rs1805087 according to the 1000 Genomes online database and present analysis.** EAS: East Asian, EUR: European, AFR: African, AMR: American, and SAS: South Asian.

Finally, we identified 12 case-control studies [16–27], involving 9986 PCa patients and 40134 controls regarding rs1805087 to evaluate its association with PCa risk (Table 1). The power of our analysis was 0.213. The distribution of genotypes among controls was consistent with the HWE in all studies except two [16,19]. The control population in all the included studies consisted of age-matched men as study participants with a normal digital rectal examination profile and serum prostate-specific antigen (PSA) values <4 ng/ml, without a known history of cancer (personal or familial). To examine representativeness of our analysis, we investigated the minor allele frequency (MAF) reported for the five main worldwide populations in the 1000 Genomes Browser: East Asian, 0.105; European, 0.173; African, 0.284; American, 0.177; and South Asian: 0.321 (Figure 2). In the same interval of the 1000 Genomes Browser database, MAFs in our analyses were 0.183 and 0.178 in the case group and control group, respectively (https://www.ncbi.nlm.nih.gov/projects/SNP/snp_ref.cgi?rs=1805087). The various genotyping methods employed in the analyzed studies included polymerase chain reaction with restriction fragment length polymorphism (PCR-FLIP), sequencing, TaqMan, GeneChip, Illumina, and SNaPshot analyses [16–27].

**Table 1:** Basic information for included studies of the association between *METH rs1805087* polymorphism sites and prostate cancer susceptibility.

Author	Year	Country	Ethnicity	Case	Control	Status	SOC	Case				Control			HWE	Genotype	NOS
								GG	GA	AA		GG	GA	AA			
Qu	2016	China	Asian	1817	2026	Published	HB	20	316	1481		15	319	1692	0.993	SNaPshot analysis	9
Cai	2010	China	Asian	217	220	Published	HB	5	27	185		3	29	188	0.139	PCR-RFLP	7
Ebrahimi	2017	Iran	Asian	100	100	Published	HB	13	53	34		6	37	57	0.998	PCR-RFLP	7
López-Cortés	2013	Ecuador	Caucasian	104	110	Published	PB	3	9	92		1	5	104	0.006	Sequencing	9
Marchal	2008	Spain	Caucasian	181	204	Published	HB	9	54	118		11	55	138	0.088	Taqman	7
Weiner	2012	Russia	Caucasian	370	285	Published	HB	15	134	221		16	96	173	0.579	Taqman	7
Stevens	2008	USA	Caucasian	794	1105	Published	PB	42	351	401		53	324	728	0.032	Taqman	9
FHS SHARe	2008	USA	Caucasian	172	231	Unpublished	HB	7	55	110		9	69	153	0.728	GeneChip	7
CGEMS	2008	USA	Caucasian	1162	1112	Unpublished	PB	48	376	738		38	340	734	0.858	Illumina	7
UKGPCS	2008	UK	Caucasian	1850	1886	Unpublished	PB	84	590	1176		71	547	1268	0.213	Illumina	7
deCODE	2008	Iceland	Caucasian	1619	30779	Unpublished	PB	60	466	1093		1044	9160	20575	0.532	Sequencing	7
ProtecT	2008	UK	Caucasian	1600	2076	Unpublished	PB	52	515	1033		84	637	1355	0.402	allele-specific PCR (KASPar) and Taqman	7

HWE: Hardy–Weinberg equilibrium; HB: hospital-based; PB: population-based; PCR-FLIP: polymerase chain reaction and restrictive fragment length polymorphism, NOS: Newcastle-Ottawa Scale

### Quantitative synthesis

**Figure 3:**
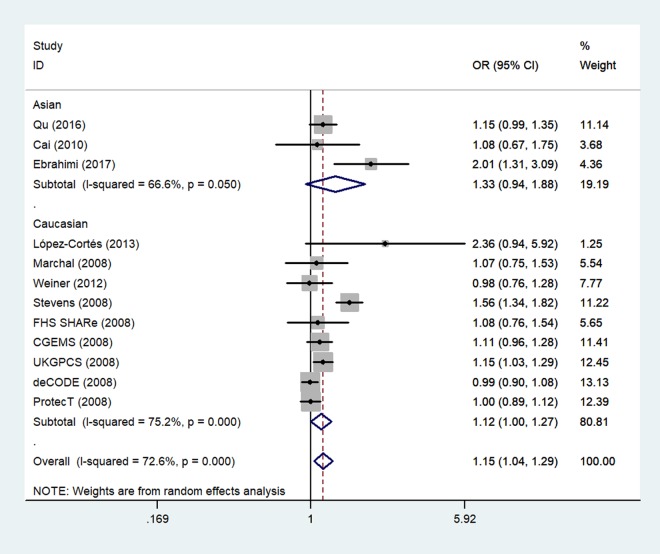
**An overall forest plot of PCa risk associated with rs1805087 (G allele vs. A allele).** The squares and horizontal lines correspond to the study-specific OR and 95% CI. The area of the squares reflects the weight (inverse of variance). The diamond represents the summary OR and 95% CI.

The overall meta-analysis results suggested significant associations between two rs1805087 genotypes and increased PCa susceptibility for all genetic models (e.g., homozygote comparison: OR = 1.15, 95% CI 1.00–1.32, *P*_heterogeneity_ = 0.263, *P* = 0.048; dominant model: OR = 1.20, 95% CI 1.05–1.38, *P*_heterogeneity_ = 0.000, *P* = 0.009; allelic comparison: OR = 1.15, 95% CI 1.04–1.29, *P*_heterogeneity_ = 0.000, *P* = 0.008; Figure 3; heterozygote comparison: OR = 1.20, 95% CI 1.04–1.38, *P*_heterogeneity_ = 0.000, *P* = 0.012; Table 2). After exclusion of the two studies where the data did not obey the HWE, the overall observed association did not change (Table 2), indicating that our analysis was powerful, stable, and representative.

**Table 2:** Total and stratified subgroup analysis for *METH rs1805087* polymorphism sites and prostate cancer susceptibility.

Variables	N	Case/	G-allele vs. A-allele		GA vs. AA		GG vs. AA		GG+GA vs. AA
		Control	OR(95%CI) *P*_h_ *P*		OR(95%CI) *P*_h_ *P*		OR(95%CI) *P*_h_ *P*		OR(95%CI) *P*_h_ *P*
Total	12	9986/40134	1.15(1.04-1.29)0.000 0.008		1.20(1.04-1.38)0.000 0.012		1.15(1.00-1.32)0.263 0.048		1.20(1.05-1.38)0.000 0.009
With HWE	10	9088/38909	1.08(1.00-1.17)0.074 0.040		1.07(1.01-1.14)0.160 0.027		1.11(0.96-1.29)0.252 0.154		1.10(1.00-1.20)0.080 0.039
Ethnicity									
Asian	3	2134/2346	1.33(0.94-1.88)0.050 0.109		1.31(0.84-2.05)0.044 0.233		1.93(1.14-3.26)0.390 0.014		1.38(0.87-2.20)0.024 0.174
Caucasian	9	7852/37788	1.12(1.00-1.27)0.000 0.052		1.19(1.01-1.40)0.000 0.043		1.10(0.95-1.28)0.462 0.181		1.18(1.01-1.38)0.000 0.042
Source of Control									
HB	6	2857/3066	1.14(1.02-1.28)0.144 0.017		1.16(1.02-1.31)0.265 0.028		1.28(0.89-1.82)0.232 0.181		1.16(1.03-1.32)0.145 0.018
PB	6	7129/37068	1.16(1.00-1.34)0.000 0.053		1.22(0.99-1.50)0.000 0.067		1.13(0.97-1.32)0.275 0.116		1.22(1.00-1.48)0.000 0.054
Genotype methods									
Others	2	1989/2257	1.14(0.99-1.31)0.747 0.069		1.13(0.96-1.32)0.931 0.135		1.37(0.79-2.40)0.582 0.265		1.14(0.98-1.33)0.865 0.090
Taqman	4	2945/3670	1.14(0.87-1.49)0.000 0.333		1.29(0.90-1.84)0.000 0.172		0.98(0.77-1.25)0.183 0.857		1.24(0.87-1.77)0.000 0.235
PCR-RFLP	2	317/320	1.49(0.81-2.74)0.058 0.202		1.50(0.60-3.73)0.026 0.385		2.78(1.19-6.50)0.404 0.018		1.61(0.65-3.99)0.020 0.307
Sequencing	4	4735/33887	1.09(0.97-1.23)0.056 0.162		1.05(0.97-1.13)0.101 0.240		1.19(0.99-1.43)0.676 0.070		1.09(0.95-1.25)0.061 0.217
Publish status									
Published	7	3583/4050	1.29(1.06-1.58)0.003 0.012		1.38(1.04-1.83)0.000 0.024		1.38(1.05-1.83)0.278 0.023		1.39(1.06-1.82)0.000 0.017
Unpublished	5	6403/36084	1.05(0.99-1.11)0.241 0.111		1.05(0.98-1.12)0.288 0.161		1.08(0.92-1.27)0.415 0.339		1.05(0.99-1.12)0.249 0.120

*P*_h_: value of *Q*-test for heterogeneity test; *P*: *Z*-test for the statistical significance of the OR; HWE: Hardy–Weinberg equilibrium; HB: hospital-based; PB: population-based; PCR-FLIP: polymerase chain reaction and restrictive fragment length polymorphism.

**Figure 4:**
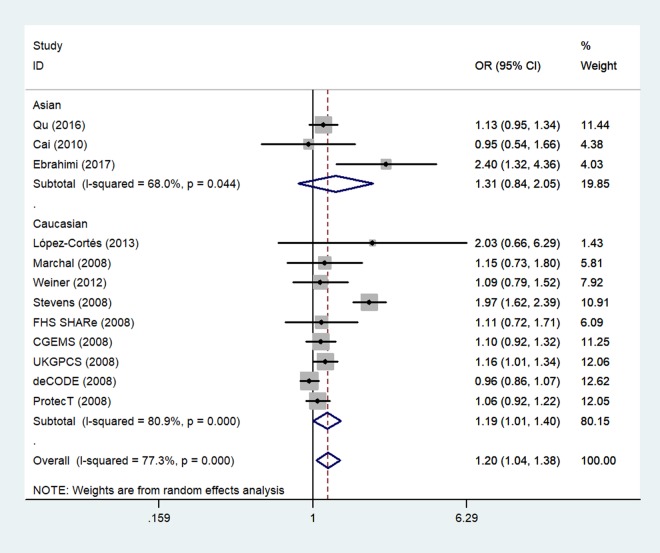
**A forest plot of PCa risk associated with rs1805087 among whites (genotype GA vs. AA).** The squares and horizontal lines correspond to the study-specific OR and 95% CI. The area of the squares reflects the weight (inverse of variance). The diamond represents the summary OR and 95% CI.

**Figure 5:**
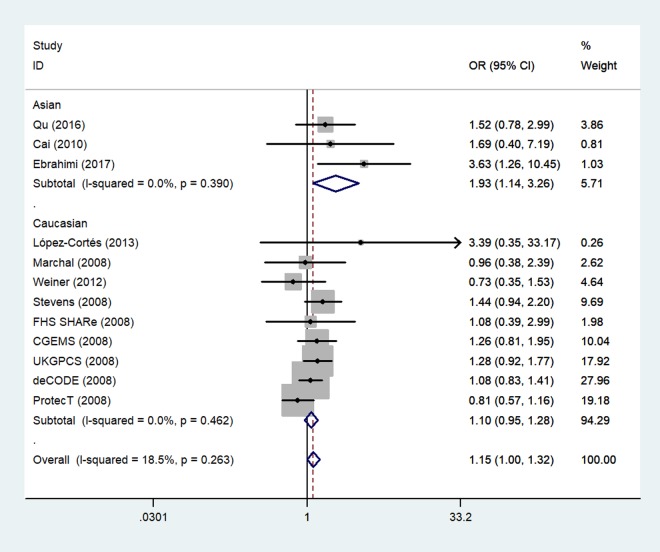
**A forest plot of PCa risk associated with rs1805087 among Asians (genotype GG vs. AA).** The squares and horizontal lines correspond to the study-specific OR and 95% CI. The area of the squares reflects the weight (inverse of variance). The diamond represents the summary OR and 95% CI.

Next, we stratified the studies by ethnicity. We found that there was a similar relation between two rs1805087 genotypes and PCa susceptibility among both Asians and whites (whites: genotype GA vs. AA: OR = 1.19, 95% CI 1.01–1.40, *P*_heterogeneity_ = 0.000, *P* = 0.043; Figure 4; GG+GA vs. AA: OR = 1.18, 95% CI 1.01–1.38, *P*_heterogeneity_ = 0.000, *P* = 0.042, Asians: GG vs. AA: OR = 1.93, 95% CI 1.14–3.26, *P*_heterogeneity_ = 0.390, *P* = 0.014; Figure 5; Table 2).

In the stratified analysis of the source of the control subgroup, significant associations were found among hospital-based controls (e.g.: *G*-allele vs. A-allele: OR = 1.14, 95% CI 1.02–1.28, *P*_heterogeneity_ = 0.144, *P* = 0.017; genotype GA vs. AA: OR = 1.16, 95% CI 1.02–1.31, *P*_heterogeneity_ = 0.265, *P* = 0.028 and GG+GA vs. AA: OR = 1.16, 95% CI 1.03–1.32, *P*_heterogeneity_ = 0.145, *P* = 0.018) but not among population-based controls (Table 2).

**Figure 6:**
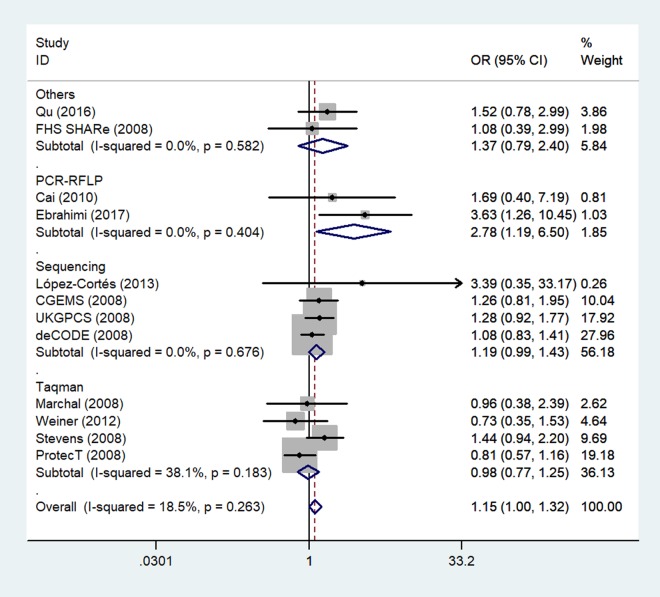
**A forest plot of PCa risk associated with rs1805087 in terms of genotype methods (GG vs. AA).** The squares and horizontal lines correspond to the study-specific OR and 95% CI. The area of the squares reflects the weight (inverse of variance). The diamond represents the summary OR and 95% CI.

Because several genotyping methods were involved, we also analyzed the genotype method subgroup. In this subgroup, we observed a significant association between rs1805087 and increased PCa susceptibility only according to the PCR-RFLP method (GG vs. AA: OR = 2.78, 95% CI 1.19–6.50, *P*_heterogeneity_ = 0.404, *P* = 0.018; Figure 6; Table 2).

**Figure 7:**
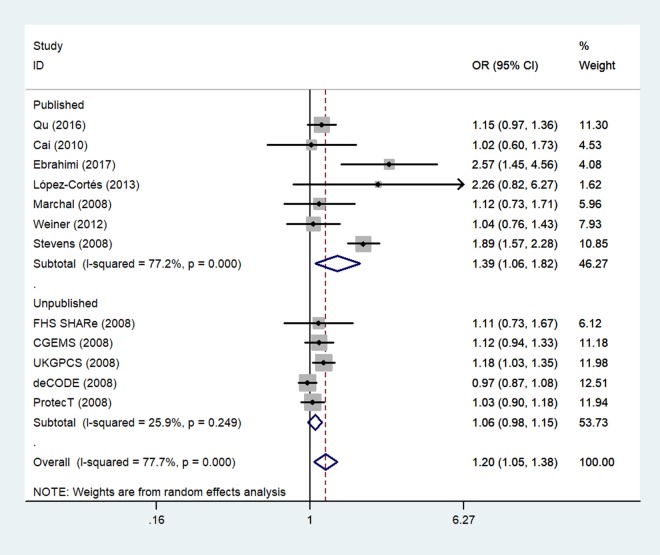
**A forest plot of PCa risk associated with rs1805087 in terms of publication status (genotypes GG+GA vs. AA).** The squares and horizontal lines correspond to the study-specific OR and 95% CI. The area of the squares reflects the weight (inverse of variance). The diamond represents the summary OR and 95% CI.

Finally, because five unpublished studies were included, we examined the publication status subgroup. Compared to the unpublished studies, significant associations between rs1805087 genotypes and increased PCa susceptibility were found in published studies (e.g., GG+GA vs. AA: OR = 1.39, 95% CI 1.06–1.82, *P*_heterogeneity_ = 0.000, *P* = 0.017; Figure 7; Table 2).

### Sensitivity analysis and bias detection

**Figure 8:**
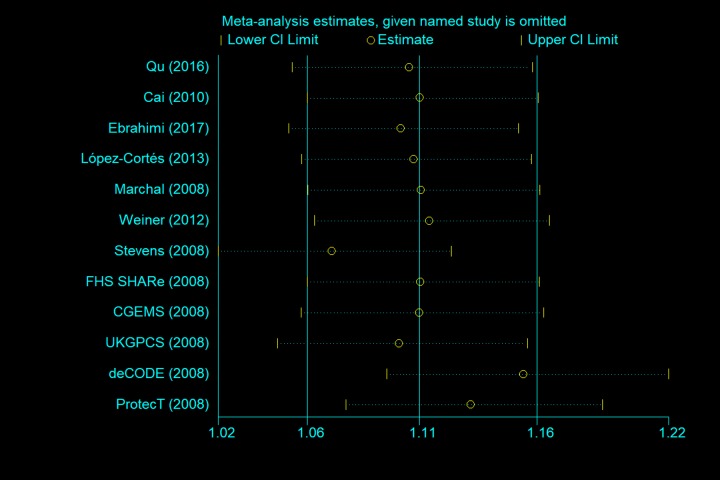
Sensitivity analysis between rs1805087 and PCa risk (G allele vs. A allele).

**Figure 9:**
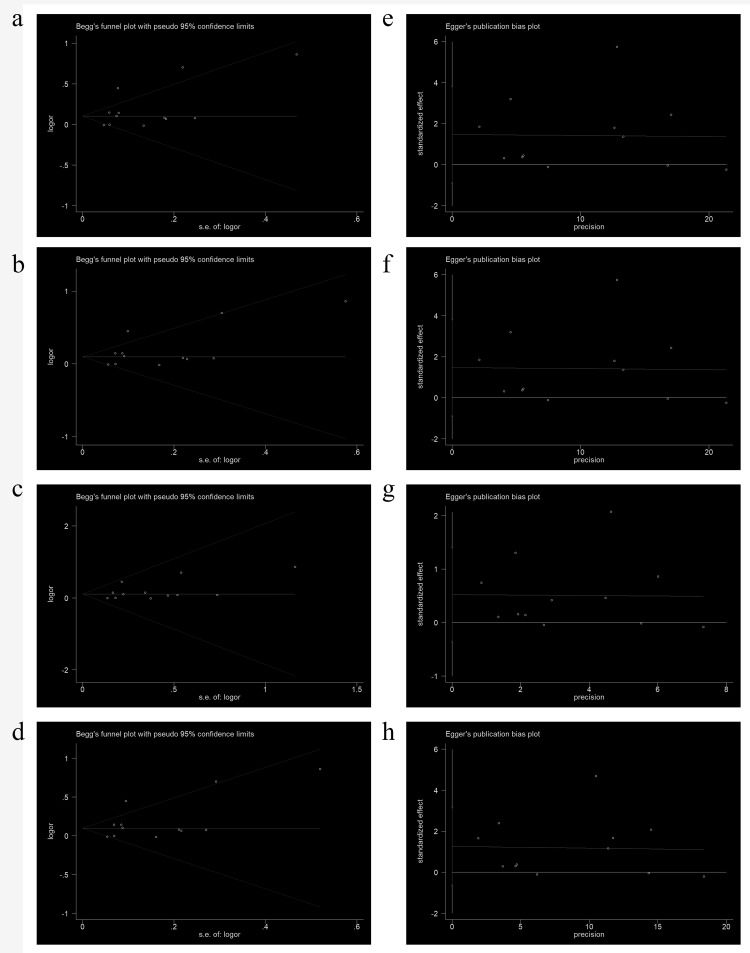
**Begg’s funnel plot for publication bias testing in analyses involving association of rs1805087 with PCa risk.** (**a**: G allele vs. A allele; **b**: genotype GA vs. AA; **c**: GG vs. AA; **d**: GG+GA vs. AA). Each point represents a separate study on the indicated association. Log [OR] stands for the natural logarithm of OR. The horizontal line indicates mean effect size. **b**: Egger’s publication bias plot of rs1805087 and PCa risk (**e**: G allele vs. A allele; **f**: genotype GA vs. AA; **g**: GG vs. AA; **h**: GG+GA vs. AA).

We next performed a sensitivity analysis on two studies [20,24], where the controls did not conform to the HWE, to determine the effect of excluded specific studies. In addition, the sensitivity analysis was carried out to determine whether modification of the inclusion criteria of the meta-analysis affected the results. According to our sensitivity analysis, no other single study (including the two studies described above) influenced the summary OR qualitatively (Figure 8). Begg’s funnel plot analysis and Egger’s test were performed to assess the literature publication bias. The shape of the Begg’s funnel plot did not reveal any obvious asymmetry, and Egger’s test ruled out any publication bias (e.g., G allele vs. A-allele: T allele vs. C allele, *t* = 1.38, *P* = 0.196 in Egger’s test; and *z* = 1.03, *P* = 0.304 in Begg’s test; Table 3, Figure 9).

**Table 3:** Publication bias tests (Begg’s funnel plot and Egger’s test for publication bias test) for *METH rs1805087* polymorphism.

Egger's test							Begg's test	
Genetic type	Coefficient	Standard error	*t*	*P* value	95%CI of intercept		*z*	*P* value
G-allele vs. A-allele	1.465739	1.059131	1.38	0.196	0.8941511- 3.825629		1.03	0.304
GA vs. AA	1.174084	0.8164867	1.4	0.181	0.6451616-2.99333		1.17	0.244
GG vs. AA	0.521603	0.3957242	1.32	0.217	0.3601251-1.403332		1.03	0.304
GG+GA vs. AA	1.25699	0.8545912	1.47	0.172	0.6471578- 3.161138		1.17	0.244
GG vs. GA+AA	0.524554	0.4058054	1.29	0.225	0.379637- 1.428745		1.03	0.304

## DISCUSSION

Identification of SNPs that affect gene expression or function and contribute to cancer susceptibility helps to predict individual and population risks as well as to understand the pathogenesis of cancer [32]. Owing to its deep involvement in cancer development, the *METH* rs1805087 SNP in several types of cancers has been widely studied: breast cancer, head and neck carcinoma, lung cancer, pancreatic cancer, gastrointestinal cancer, non-Hodgkin lymphoma, and ovarian cancer, to name a few [33–37]. No significant association has been detected between rs1805087 and any cancer types mentioned above.

Kimura et al. [30] were the first to report an association between rs1805087 and increased PCa risk. Nonetheless, the allele distribution of rs1805087 did not differ among PCa patients stratified by age, clinical stage, or the presence of metastases. To our knowledge, sites with altered DNA methylation are found in many human cancers, including those prevalent in PCa. The enzymes methylenetetrahydrofolate reductase (MTHFR), METH, METH reductase (METHR), and thymidylate synthase perform essential functions in folate metabolism: an important source of materials for DNA and RNA synthesis and methylation. The remethylation of homocysteine to methionine is catalyzed by METH in a vitamin B12–dependent reaction (B12 acts as an intermediary carrier of a methyl group). METH becomes inactive upon oxidation of the remethylation cofactor (vitamin B12) by METHR. The latter catalyzes the regeneration of methylcobalamin, a METH cofactor, important for keeping METH active [38,39]. Rs1805087, one of the valuable SNPs in relation to PCa risk, may increase METH expression and indirectly participate in DNA methylation and the folate metabolic pathway. Subsequently, several investigators have confirmed these findings in larger samples and different populations [18,23,25]. Nonetheless, the lack of systematic and cohesive analysis renders these results ambiguous, even within the same population.

Meta-analysis is a means of increasing the effective sample size by pooling data from association studies, thereby enhancing the statistical power of estimation of genetic effects [40]. In 2009, Collin et al. [16] reported the first relevant meta-analysis, which included eight case-control studies (five were unpublished studies) and found no association between rs1805087 and PCa risk in all genetic models. By contrast, a significant association was observed between rs1805087 in localized increased PCa susceptibility (dominant model comparison, OR = 1.16, 95% CI 1.04–1.29, *P* = 0.008). A growing number of original research articles and unpublished studies makes it necessary to combine data from all the studies to date and to reanalyze the association between rs1805087 and PCa risk.

In this study, we analyzed the associations between genotypes of rs1805087 and PCa risk by the meta-analysis method to reach a statistically backed conclusion. The potential ability of rs1805087 to increase PCa risk was investigated in a sample of 9986 cases and 40134 controls from seven published and five unpublished case-control studies. The conclusions from our results are 1) individuals who carry the rs1805087 G allele have increased susceptibility to PCa; 2) rs1805087 may increase PCa risk among both Asians and whites. These conclusions are reported for the first time and warrant continuation of research into rs1805087 regarding other types of cancer.

Of the 12 studies, excluding the five unpublished studies [16,17,19,22,26], six detected no significant association between rs1805087 and PCa risk. Only one study [27] indicates that the AG genotype, GG genotype, and G allele are associated with higher PCa risk. Nonetheless, in our meta-analysis of risk estimates, when all the studies were pooled, we obtained evidence for a significant association between rs1805087 and higher PCa risk. This finding can be explained as follows: first, the etiology of PCa is complicated, involving gene–gene and gene–environment (including gene–nutrition) interactions. Therefore, the environmental (including nutritional) factors cannot be ignored. Future studies that combine other genes from folic acid and methionine metabolic pathways with METH are needed for accurate analysis and interpretation. Furthermore, if the number of included studies had not been large, false negative results may have been obtained about this SNP. Thus, further studies should have a larger sample size. It would be beneficial to use meta-analysis to obtain more accurate results.

Although we made considerable efforts and devoted substantial resources to testing the possible association between rs1805087 and PCa risk, there are some limitations inherent in the included studies. First, despite inclusion of all the eligible studies, the resultant sample size is still not large enough [20,27]; this situation may increase the likelihood of type I and type II errors. Second, the literature review was primarily based on PubMed and Embase databases. Thus, some publications may be missing, thereby causing some bias in risk estimates. Third, the lack of original data, such as age, gender, the body–mass index, diet, alcohol consumption, smoking status, and a family history of cancer, limited our ability to further evaluate the adjusted OR and gene–environment interactions. Therefore, it is necessary to evaluate the roles of some special environmental factors, including lifestyles. Fourth, moderate heterogeneity was observed in overall comparisons and several subgroups; this heterogeneity could result from small sample sizes and from differential effects of the analyzed SNP in different ethnic groups involved. Fifth, we included five unpublished studies from Collin et al. (2009). The limitations of using unpublished studies are a) frequencies in these studies are not strictly evaluated by a peer review process, and b) the results of these studies may confuse the results of published studies. Besides, we included all the published and unpublished studies in our current analysis to show that this is a comprehensive meta-analysis and may yield powerful and objective conclusions. The data of these five unpublished studies all came from a published study (Collin et al., 2009) and therefore were not unpublished strictly speaking and may be considered a valid source and can increase the credibility of conclusions.

In summary, our meta-analysis shows that *METH* SNP rs1805087 may increase susceptibility to PCa. Further well-designed studies with a large sample size, different ethnicities, and detailed environmental factors are needed to validate the conclusions of our meta-analysis.

## MATERIALS AND METHODS

### The search strategy

We searched the PubMed and Embase databases for all articles on the association between rs1805087 and PCa risk up to January 20, 2018. The medical subject headings and key words used for the search were “MTR or methionine synthase,” and “prostate cancer or carcinoma,” and “polymorphism or variant.” The electronic search also covered the reference lists of all the identified articles and reviews to find possible additional original reports. All the included studies met the following criteria: (1) the design was “case-control study,” (2) the association between rs1805087 and PCa risk was explored (both published and unpublished), and (3) carcinoma cases were diagnosed histopathologically. The major exclusion criteria were (1) duplicate data; (2) abstract only, commentary, review, or editorial publications; and (3) insufficient reporting of data.

### Data extraction

The following information was collected: the first author’s last name, year of publication, country of origin, ethnicity, source of the control (hospital-based or population-based) and the Hardy–Weinberg equilibrium (HWE) of the control, total number of cases/controls, and genotyping method. Subgroups were classified based on the source of the control, on ethnicity, genotyping method, and publication status.

### Quality score assessment

The Newcastle-Ottawa Scale (NOS) [41] was selected to assess the quality of each study. This measure assesses aspects of the methodologies used in observational studies, which are related to the study quality, including selection of cases, comparability of populations, and ascertainment of exposure to risks. The NOS rating ranges from zero stars (worst) to nine stars (best). Studies with a score of seven stars or greater was considered as a high quality.

### Statistical analysis

The strength of the association between rs1805087 and PCa risk was measured by odds ratios (ORs) with 95% confidence intervals (CIs). Pooled ORs were obtained from a combination of studies by heterozygote comparison, homozygote comparison, dominant and recessive models, and allelic comparison. Heterogeneity among the studies was checked using the χ^2^-based *Q* statistic and was considered statistically significant at *P* < 0.10 [42]. At *P* > 0.10, the pooled OR of each study was calculated via the fixed-effects model (the Mantel–Haenszel method, which weights the studies by the inverse of the variance of estimates). Otherwise, the random-effects model (the DerSimonian and Laird method) was used [43,44]. The significance of the pooled OR was determined by the *Z*-test, and data with *P* < 0.05 were considered statistically significant. The departure of frequencies of rs1805087 from the expected values under the HWE was assessed by the χ^2^ test among controls; *P* < 0.05 was assumed to denote a significant disequilibrium. Publication bias was detected with Egger’s linear regression method and funnel plot; the funnel plot asymmetry was assessed by Begg’s test; data with *P* < 0.05 were considered statistically significant [45]. All statistical tests in this meta-analysis were two-sided and performed in the STATA software, version 10.0 (STATA Corp., College Station, TX, USA). The power of our meta-analysis was calculated by means of software called PS: Power and Sample Size Calculation (http://biostat.mc.vanderbilt.edu/wiki/Main/PowerSampleSize#Windows) [46].

## Supplementary Material

PRIZMA 2009 Checklist
